# Feasibility results from a randomized trial of a text message–delivered sexual violence harm reduction intervention among college students

**DOI:** 10.21203/rs.3.rs-7367961/v1

**Published:** 2025-11-03

**Authors:** Jocelyn Anderson, Lakyn Webb, Wendy Huynh, Miranda Ortega, Whitney Norris, RaeAnn Anderson

**Affiliations:** University of Arkansas for Medical Sciences; University of Arkansas for Medical Sciences; The Pennsylvania State University; University of Arkansas for Medical Sciences; University of Arkansas for Medical Sciences; University of Missouri-Kansas City

**Keywords:** sexual assault, alcohol use, intervention, text message, college campus

## Abstract

**Objectives:**

We conducted a Stage 1B pilot randomized controlled trial in which a text message was used to reduce the degree of harm caused by sexual violence. The complete study protocol was assessed to inform future intervention delivery and research processes for a full-scale trial.

**Methods:**

This feasibility randomized trial compared two text message-delivered intervention conditions: alcohol reduction content and sexual violence harm reduction content. We recruited college students aged 18–24 years who reported recent (past 30 days) binge drinking to participate in the 12-week intervention and to provide self-reported data on outcomes prior to randomization, postintervention, and 3 months after intervention completion. *A priori* feasibility metrics, including recruitment, retention, intervention participation, and missing data, were assessed.

**Results:**

A total of 183 college students were randomized to one of the intervention conditions and participated in the study. Two of the four feasibility measures were met (retention and missing data), whereas two underperformed their goals (proportion of messages responded to and enrollment of eligible screened individuals). As anticipated, no statistically significant outcome findings were noted, as we were underpowered for the measured outcomes.

**Conclusions:**

While the enrollment of eligible screened participants was lower than the goal, we were still able to recruit over 180 of the target 200 participants, and a 98% retention rate made up for the lower than anticipated recruitment. Data regarding the details of which text messages participants were most or least likely to engage with and why presented opportunities to refine the intervention prior to full-scale testing.

## Background

Sexual violence is a significant public health issue globally.^1^ Sexual violence, as defined by the Centers for Disease Control and Prevention, is “any sexual act attempted or committed that is not consensual or freely given by the victim or against someone who is unable to give consent or refuse.”^2^* Although often unreported, approximately one-third of men and over half of women in the United States have reported experiences of sexual violence.^3^ Recent estimates illustrate the costliness of sexual violence. It is estimated that rape incurs costs of $122,461 per survivor, including healthcare services, productivity loss, and legal costs.^3^ Research has shown that marginalized and minoritized people experience sexual violence at even higher rates. This has been documented among people identifying as lesbian, gay, bisexual, and transgender; racial and ethnic minority groups; people with disabilities; and immigrants and refugees.^3, 4, 5–7^

*Footnote: We recognize that many people who have experienced sexual violence do not identify with the term “victim”.^8^ In this paper, we use this term only when specifically quoting or referencing work that uses the term.

While sexual violence is one of the most commonly experienced crimes on college campuses, fewer than 30% of students report their experiences of sexual violence to any formal authority (e.g., police, Title IX, health care provider, advocate).^9^ Alcohol use (on the part of the victim, perpetrator, or both) is a frequently cited reason that students provide for not seeking services, including reporting to law enforcement, following sexual violence^9^. However, more than 75% of rapes on college campuses include alcohol.^10^

In recent years, the proliferation of cell phones and the COVID-19 pandemic have spurred an increase in text messages and app-based behavioral health interventions^11–13^. These digital interventions leverage the ubiquity of cellular devices to deliver behaviorally tailored text message interventions directly into individuals’ daily lives, providing support and promoting behavioral change in real time^14^. For example, young adults with hazardous drinking behaviors have demonstrated reduced binge drinking days after engaging with text message-based interventions, highlighting the potential of this approach for addressing risky behaviors over short time frames^14^.

Similarly, efforts to prevent sexual violence on college campuses have evolved to include various delivery methods. Traditional approaches range from intensive multisession interventions designed to transform attitudes and behaviors to minimal online modules aimed at fulfilling legal requirements for information dissemination^15–17^. Emerging interventions are now utilizing text messages and other digital technologies to deliver just-in-time information, support, and goal-setting for the prevention of sexual violence^18^. These innovative approaches provide an opportunity to extend the reach and impact of sexual violence prevention strategies, particularly in contexts where traditional methods may face barriers to accessibility or engagement. These accessibility issues are particularly prevalent in minority populations^19. 20^.

Addressing alcohol use and sexual violence together is a promising approach for improving safety and reducing harm among college students. Alcohol use and sexual violence are often cooccurring phenomena, yet they are traditionally treated in silos, which can limit the effectiveness of prevention efforts^21, 22^. We acutely recognize that alcohol use is not the root cause of sexual violence in society.^25, 24^ However, we also recognize the importance of creating interventions that must live in the current structures.^25^ Recognizing this gap, we utilized qualitative interviews and focus groups to develop theory-based content aimed at addressing alcohol-related sexual violence and promoting harm-reduction behaviors among college students. This just-in-time intervention provides real-time information, goal-setting reminders, and goal feedback that can reach students in high-risk situations. Unlike traditional educational programs, which are typically offered only at the beginning of the academic year, this multitarget intervention aims to achieve two key outcomes: increasing students’ knowledge of alcohol-related sexual violence definitions and risks through informational reminders and encouraging the adoption of harm-reduction behaviors, such as reducing alcohol consumption through goal-setting prompts and responses modeled after motivational interviewing methods.^21^ The rationale for this approach stems from evidence suggesting that interventions addressing both alcohol use and sexual violence simultaneously may be more effective than those targeting these issues separately.^26,27^ Research has shown that text message interventions are particularly well suited for behavior change, as they allow for tailored, just-in-time delivery of information and reminders, which can reinforce knowledge and promote sustained behavioral shifts.^14,27^ Moreover, integrating these topics into a single intervention acknowledges the interconnected nature of these risks and may reduce the fragmented delivery of campus prevention efforts and provide more time-efficient intervention efforts. To evaluate this novel approach, the Stage 1B pilot study was designed to assess the feasibility of both reducing the degree of harm caused by sexual violence and the research procedures required for its testing.^28^ This integrated strategy represents an important step toward addressing alcohol use and sexual violence as interconnected public health issues, with the potential to increase the safety and well-being of college students through innovative and accessible intervention methods.

## Methods

### Overview

We conducted a Stage 1B pilot randomized controlled trial of a text message delivered intervention. While this pilot trial was not powered to detect differences between groups in all of the relevant clinical outcomes, we piloted the complete study protocol and aimed to use these data to adjust the intervention delivery and research process as necessary for a full-scale trial.^30^ All study procedures were reviewed and approved by the Institutional Review Boards at Penn State University (STUDY00010873) and the University of Arkansas for Medical Sciences (275726). The study was also registered at ClinicalTrials.gov (NCT0506591).

#### Recruitment and Enrollment

Between February 2022 and January 2023, we recruited participants via flyers, social media (e.g., Instagram, Facebook), and word-of-mouth and studied information sharing via campus networks (e.g., the campus Gender Equity Center and Center for Sexual and Gender Diversity). The recruitment materials directed the students to the study website, which outlined the study information, an online screening survey, and a brief consent process. The inclusion criteria were as follows: 1) aged 18–24 years, 2) currently enrolled as a college or university student enrolled in either an undergraduate or graduate program, 3) owned a mobile phone with internet access and an unlimited text message plan, 4) reported binge drinking in the past 30 days (four or more drinks for women or five or more drinks for men^31^), and 5) were able to participate in English. Eligible students were shown a study consent information sheet on screen to review and agree to or decline.

Students who completed the screening and agreed to participate were placed into a “to be enrolled” queue and followed up with by a member of the research team. To provide informed consent, understand the elements of participation, and minimize the risk of fraudulent enrollment, potential participants were required to complete a brief phone call with a member of the research team before being enrolled in the study. During this call, the participants completed a verbal consent process. Once the enrollment call was completed, each participant received a unique baseline survey link to complete. Following the completion of the baseline survey measures, the participants were automatically computer randomized into either the intervention or control condition. We utilized simple randomization. Neither the participants nor the researchers were blinded because the nature of the intervention was the receipt of messages.

#### Control conditions

The control condition included a previously developed text message-delivered alcohol use reduction intervention.^14,31–33^ This condition asked participants to commit to a goal, i.e., “Would you be willing to set a goal to drink less than X drinks when drinking?” The proposed goals were tailored on the basis of the number of drinks reported in the past week by the participants. Each week, participants who were willing to set a goal received a goal reminder and a goal met (“Another week of no alcohol is another week of setting yourself up for great health”) or not met (“You may wish to think about how you got into this situation to avoid it in the future”) feedback text message. The messages attempted to reinforce goal successes and reframe goal failures. If the participant did not set a goal, the participant received feedback on alcohol quantity each week (e.g., “FACT: Having more than 3 drinks can result in a loss of motor coordination for up to 12–18 hours after drinking.” or “It is OK to have mixed feelings about reducing your drinking.”).

#### Intervention conditions

Each week, participants received a randomly selected message from the message library that addressed one of the following: 1) setting a safety goal prior to planned drinking events (e.g., “Do you have a plan for getting home tonight? “), 2) reminders about harm reduction strategies (e.g., “Be aware of what’s in your drink (premade drinks & jungle juice). When in doubt, stick to what you know.”), 3) reminders about how to access services on campus if someone experiences sexual violence. Each Sunday, a structured end-of-weekend check-in was held to assess the students’ alcohol content (“This week, what is the greatest number of drinks you had on any drinking occasion? (round to the closest whole number 0, 1, 2, 3…, etc., please”) and harm reduction strategy use (“Use this survey to tell us about your weekend: [LINK to list of harm reduction strategies]”). The message library provided goal reminders and feedback that were curated on the basis of qualitative interviews that included students’ feedback on the text message intervention focused on harm reduction^21^. Prior to study initiation, we piloted our text message-delivery process. We used Twillio to deliver automated text message intervention content over a 12-week period, with 25 students meeting the same eligibility criteria as in this study. Twillio interfaced with the study database to allow tracking of the number of messages sent and received. All 25 of these students received the intervention content, and their data were not included in these analyses.

#### Data collection

Survey data were collected via secure internet-based surveys at baseline, immediately after completing the 12-week-long intervention (“postintervention”), and 3 months after intervention completion. Survey links were sent to participants via text messages and followed up via email if participants did not complete the surveys within a two-week window. The participant surveys were open for six weeks prior to closing and were marked as missing. Compensation in the form of Amazon.com claim codes was provided to participants via text messages following each survey completion in an escalating incentive manner: baseline $20; postintervention $30; and 3-month follow-up $50.

### Measures

The demographic variables collected for this study included age, sex, sex assigned at birth, current residence, and participation in athletic and Greek life activities.

#### Feasibility Measures

For this pilot trial, we collected several measures of feasibility of the intervention and research protocol with predefined targets: 1) proportion of people who screen as eligible for the study who completed enrollment (≥ 75%); 2) retention rates of participants after enrollment (≥ 85%); 3) rate of missing or unusable survey data (≤ 5%); and 4) proportion of text message prompts responded to (≥ 75%).

#### Outcome Measures

Our primary outcomes were as follows: 1) alcohol use and 2) use of strategies to reduce the degree of harm caused by sexual violence. Our secondary outcomes included 1) knowledge of sexual violence and alcohol risk behaviors and 2) self-efficacy in obtaining sexual consent. Our exploratory outcome was the incidence of sexual violence victimization. All outcomes were measured at postintervention and at the 3-month follow-up.

### Alcohol use

Alcohol use was measured via a 30-day timeline following the back method, in which participants were presented with a calendar for the 30 days prior to the date they were taking the survey and were asked to indicate the number of drinks they had consumed on each of the past 30 days.^34,356^ We also used this 30-day calendar to calculate the number of binge drinking ays in the past month. Binge drinking days were defined as any drinking day where a participant assigned male sex at birth reported ≥ 5 drinks in one day or a participant assigned female sex at birth reported ≥ 4 drinks in one day.^31^

### Use of strategies to reduce the degree of harm caused by sexual violence

The use of strategies to reduce the degree of sexual violence harm was assessed with an 11-item protective behavior scale from the American College Health Association’s National College Health Survey.^36^ Participants reported how often when drinking/socializing in the past 3 months, they used each of the specific behaviors (e.g., “Stay with the same group of friends the entire time you were drinking” or “Have a friend let you know when you have had enough”) on a Likert scale ranging from “never” to “always” (observed baseline ∝ = 0.73).

### Knowledge of sexual violence and alcohol risk behaviors

We measured knowledge of alcohol-related sexual violence via the 12-item Alcohol and Sexual Consent Scale ∝ = 0.76.^37^ This tool included items such as “A woman who is drinking heavily can still provide legal consent for sexual activity” and “Alcohol use makes a person more vulnerable to sexual assault,” which assess knowledge of alcohol-related sexual violence and the risk of sexual violence. The response options on a 5-point Likert scale range from strongly disagree to strongly agree. The responses are totaled (including 6 reverse-coded items) to calculate a score ranging from 12 to 60 (observed baseline ∝ = 0.24). The sample used to measure the reliability of this measure was made up of first-year college students, whereas the current study primarily included second-year through fourth-year college students, which could have contributed to a more diverse knowledge base.

### Self-efficacy to obtain sexual consent

The 11-item Sexual Consent Scale - Revised^38, 39^ was used to assess self-efficacy to obtain sexual consent (observed baseline ∝ = 0.92). This scale asked participants to rate their agreement (from Strongly Disagree to Strongly Agree) with items such as “I feel confident that I could ask for consent from a new sexual partner” and “I always verbally ask for consent before I initiate a sexual encounter.” Items were summed to a theoretical range between 11 and 55. A higher total score indicates greater self-efficacy in obtaining sexual consent.

### Experiences of sexual violence

While not a primary outcome of this pilot trial, we also explored experiences of sexual violence via the Sexual Experiences Survey^40^. The participants were asked how many times (on a 5-point Likert scale ranging from 0 to 4 or more) each of the six sexual violence items had occurred in the past 3 months. At baseline, participants were also asked the same series of items and if they had happened at any point prior to starting college. The responses to the six sexual violence items were dichotomized into yes or no for any reported experience of any sexual violence in the past 3 months at each timepoint.

### Sample size

A feasible sample size of 200 was determined on the basis of data from prior work performed in college health centers^41^. In *a priori* power analysis, we determined that we would not have sufficient power to detect statistically and clinically significant results from this pilot trial. For our two anticipated primary outcomes in a full-scale trial (alcohol use and use of sexual violence harm reduction strategies), our calculations revealed 820 participants (410 per arm) and 2,504 participants (1,202 per arm), respectively, to achieve 80% power at ∝=0.05, assuming a within-subject correlation of 0.4 between baseline and follow-up assessment.

### Data analysis

**Figure 1:**
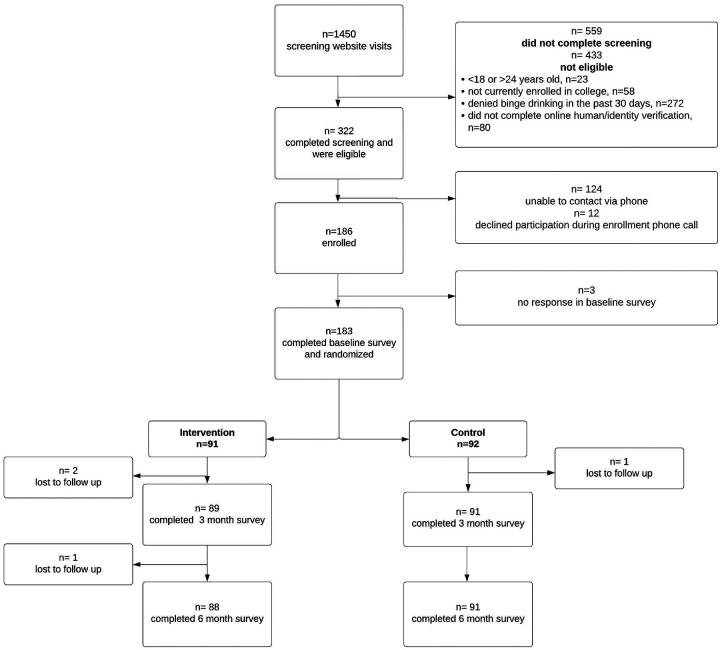
Participant Enrollment

In addition to descriptive statistics assessing feasibility goals (see Fig. 1), we also conducted exploratory analyses of the proposed outcomes for a full-scale trial. 1) number of drinking days per month, 2) number of binge drinking days per month, 3) use of sexual violence harm reduction strategies, 4) knowledge of sexual violence and alcohol risk behaviors, and 5) self-efficacy in the use of sexual violence harm reduction behaviors. For each outcome, we tested for differences between the intervention and control conditions at 3 months and 6 months via generalized linear modeling. Each model was adjusted for age, history of sexual violence at baseline, and sex assigned at birth. Following intention-to-treat principles, analyses were conducted on all individuals who were randomized to a condition regardless of the degree to which they interacted with the intervention.

Finally, we explored whether gender or sexual violence history moderates the relationship between the intervention and each of the outcomes by repeating the primary models and including an interaction term between the intervention and gender or between the intervention and sexual violence history (in separate models). Owing to the sample size of this pilot study, we did not expect to be powered at ∝=0.05; thus, interaction terms at p < 0.2 are worth exploring for these analyses. Because our postintervention and 3-month follow-up retention rates were 98.4% and 97.8%, respectively, and missing data were < 2% for any outcome variable, we did not conduct any imputation for missing data.^42^ All the data were analyzed via SPSS.^43^

## Results

### Feasibility

A total of 1,450 students visited the screening website, with 559 (38.6%) completing the screening. Among those who completed screening, 332 were eligible (57.6%), and 186 (33.3%) were included in the study (Fig. 1). This percentage was lower than the preset goal of 75% for people who initiated enrollment and completed screening.

The study demonstrated strong feasibility for retention, exceeding its retention target and maintaining a high level of participant engagement. Specifically, 98.4% (n = 180) of the participants completed the postintervention survey, and 97.8% (n = 179) completed the 3-month survey, surpassing our target retention rate of > 85% at both time points. Of the three participants who missed the postintervention follow-up, two returned to complete the 3-month follow-up, resulting in only five participants missing one follow-up and one participant missing both.

For all outcome measures, the study targeted a missing data rate of < 5% among participants who completed the survey. The target was substantially exceeded, with actual missing data rates of < 2% for each outcome measure at postintervention and 3-month follow-ups.

Challenges with intervention delivery were minimal. Three participants (1.6%) were unable to receive text messages throughout the process. Two participants opted out by replying “STOP”, and one was excluded from the message-related analyses because of an incorrectly formatted phone number during the enrollment phone call. Despite these issues, all three participants remained in the study and completed the follow-up surveys. No adverse events were reported during the course of the study as a result of the intervention.

Individual participants responded to text message prompts between 0% and 63% of the time (median: 40%, IQR: 33–50%). Because of the nature of the intervention, the intervention condition initiated more text message encounters with participants in the intervention condition (38 v 23, p < 0.001; combined range 22–46). More messages were sent from the system to patients in the intervention condition (122 v 67, p < 0.001; combined range 6–373). Both groups replied with a similar number of inbound messages to the system (31 vs 29, p = 0.329; combined range 0–55).

### Descriptive Statistics

Among those enrolled, 183 (98.4%) completed the baseline survey and were randomized into either the intervention (n = 91) or control (n = 92) group (see Fig. 1). Among the 183 participating students, the majority identified as white/Caucasian (79.8%) or straight/heterosexual (67.8%). The majority of participants were cisgender women (73.8%), with cisgender men making up 22.4% of the sample and gender diverse students making up the remaining 3.8%. With respect to victimization history at baseline, 45.9% of the participants reported experiencing sexual violence before college, whereas 54.1% did not.

### Outcomes

#### Alcohol use

Neither the overall number of drinking days nor the number of binge drinking days in the past 30 days significantly changed at postintervention or 3 months (Table 2). Postintervention adjusted models revealed a nonsignificant decrease in the number of days (drinking days: *β* = −0.302, 95% CI: −1.669, 1.065; binge drinking days *β* = −0.371, 95% CI: −1.185, 0.444). At 3 months, the results revealed no change in the overall number of drinking days (b = − 0.007, 95% CI: −1.159, 1.144), whereas binge drinking days showed a similar nonsignificant reduction of 0.3 days (β = −0.311, 95% CI: −1.034, 0.413).

#### Use of strategies to reduce the degree of harm caused by sexual violence

The use of sexual violence harm reduction strategies was not significantly greater in the intervention group than in the control group at both the postintervention (*β* = 0.331, 95% CI: −0.317, 0.977) and 3-month follow-ups (β = 0.461, 95% CI: −0.184, 1.107).

#### Knowledge of sexual violence and alcohol risk behaviors

The participants in the intervention group demonstrated increased knowledge of alcohol-related sexual violence postintervention (*β* = 0.556, 95% CI: 0.109, 1.002). However, this effect diminished by 3 months (*β* = 0.253, 95% CI:−0.261, 0.766), highlighting the challenge of sustaining knowledge gains over time.

#### Self-efficacy in obtaining sexual consent

Self-efficacy to obtain sexual consent improved in the intervention group, but these increases were not statistically significant at either the postintervention (*β* = 0.636, 95% CI: −0.160, 1.433) or 3-month (*β* = 0.529, 95% CI: −0.304, 1.361) follow-up.

#### Incidence of sexual violence victimization

Although the odds of experiencing sexual violence decreased in the intervention group at both postintervention (OR: 0.585, 95% CI: 0.299, 1.147) and 3 months (OR: 0.819, 95% CI: 0.455, 1.473), these reductions were not statistically significant.

#### Moderation analyses

To assess the potential differential effects of the intervention, moderation analyses were performed to examine whether gender or prior history of sexual violence influenced the intervention outcomes. The results indicated no significant moderation effects for either variable on alcohol use, harm reduction strategies, or self-efficacy in obtaining sexual consent.

## Discussion

This pilot study demonstrated that we were able to recruit and engage students successfully for an integrated intervention targeting alcohol use and the reduction of harm from sexual violence. Our ability to retain a high percentage of participants (98.4% at postintervention and 97.8% at 3 months) supports the feasibility of this work. The high retention of participants at the completion of the intervention and the three-month follow-up suggest high acceptability and potential impact of this intervention. While our target for the enrollment of eligible individuals was 75%, our actual percentage was 56%. The use of a two-step (internet-based first and phone second) enrollment process allowed us to have the reach necessary to nearly meet our enrollment target (200 participants) and ensure one-on-one contact with each enrolled participant prior to beginning the study despite this lower than the anticipated number. Our high retention rate further compensated for the overall difference in total sample size (85%, 98%).

However, the engagement levels with text message prompts warrant closer examination. While participants received and responded to a significant number of messages, further research is needed to better understand specific factors influencing engagement, particularly in terms of message content and response triggers. One limitation of the study was our inability to fully integrate the alcohol use reduction content with the sexual violence harm reduction intervention given the greater number of messages that would be sent to any individual participant in the course of a week than the number of messages students indicated that they would be interested in receiving from a health-related intervention.^21^ This resulted in a comparison between alcohol use reduction messages and sexual violence content rather than a combined intervention. Future studies should continue to explore ways to combine alcohol use and sexual violence in robust and meaningful ways, as we hypothesize that such an integrated intervention could yield more comprehensive changes in behavior.

Compared with the control, the sexual violence harm reduction intervention triggered more message events and sent more total messages; however, this did not translate into higher response rates to messages from participants. This may be attributed to the fact that a larger proportion of the harm reduction messages did not require a direct response, [i.e., “Be aware of what’s in your drink (premade drinks & jungle juice) When in doubt, stick to what you know.” and “Do not forget to bring your keys with you when you go out!”] We do not know how these reminder messages, compared with messages that included interaction, may have influenced knowledge or behavior differently. Additionally, we were not able to track text message views or participants who may have blocked the study phone number from their devices. Moving forward, message prompts should be carefully evaluated to reduce response fatigue, with consideration given to creating a set of content that prompts participant thought and provides necessary information. Our qualitative findings indicated that harm reduction messages were most impactful when tailored to specific locations and times on the basis of real-world drinking behaviors.^21,46^ In this pilot, content was created specific to one campus. Adapting these prompts to be modifiable and context sensitive in future iterations of the intervention would allow for widespread implementation. Other research supports the idea that personalized interventions are more effective for young adults than are more generalized messaging approaches.^45–47^ While outside the scope of this pilot, working with artificial intelligence programs or chat bots to continue refining intervention content and delivery may provide opportunities for tailoring content to users on the basis of a larger number of factors in real time.^48, 49^

### Limitations

The use of the Twilio platform for message delivery poses challenges for tracking participant engagement. Twilio allows for randomization of message content and delivery times from the message library and complex programming of multiple message response options when needed. However, it was difficult to capture engagement with the intervention since not all messages required a response. Future studies should explore alternate systems and methods for tracking engagement and ensure that message content remains interactive and relevant to the participant’s immediate context. The pilot nature of this trial means that our sample size was not powered to detect differences in our selected outcomes. While we delayed recruitment for the study until local governmental policies regarding COVID-19 had relaxed and campuses had returned to person status, we recognize that aspects of student life, such as alcohol use and relationships, were impacted in multiple ways that we may not have accounted for in a study designed in a per-COVID-19 environment.

## Conclusion

This pilot study provides valuable insights into the feasibility of using just-in-time text message interventions for alcohol and sexual violence harm reduction among college students. By refining the message delivery system and continuing to integrate alcohol reduction and sexual violence harm reduction strategies, future research could generate more meaningful behavioral changes and offer campuses with more viable interventions for addressing these issues.

## Supplementary Material

Supplementary Files

This is a list of supplementary files associated with this preprint. Click to download.

- PilotPaperCONSORT2025editablechecklist.docx
- SupplementaryTable.xlsx

## Figures and Tables

**Table 1:** Participant Demographics at Baseline

	All *n* = 183		Intervention *n* = 91		Control *n* = 92		
	*n*	*%*	*n*	*%*	*n*	*%*	*p*
**Race**							0.982
Asian	20	10.9	10	11.0	10	10.9	
Black	5	2.7	3	3.3	2	2.2	
Multi-racial	10	5.5	5	5.5	5	5.4	
Other	2	1.1	0	0	2	2.2	
White	146	79.8	73	80.2	73	79.3	
**Year in College**							0.256
First	11	6.5	5	5.5	6	6.5	
Second	48	25.8	26	28.6	22	23.9	
Third	58	31.7	22	24.2	36	39.1	
Fourth	52	28.5	28	30.8	24	26.1	
Fifth or higher	3	1.6	3	3.3	0	0	
Graduate or professional school	11	5.9	7	7.7	4	4.3	
**Sorority or Fraternity Participation**							0.434
Yes	35	18.8	20	22.0	15	16.3	
No	148	81.2	71	78.0	77	83.7	
**Cumulative GPA**							0.766
A	110	59.7	53	58.2	57	62.0	
B	64	35.5	34	37.4	30	32.6	
C	9	4.8	4	4.4	5	5.4	
**Born in the United States**							0.426
Yes	171	6.5	83	91.2	88	95.7	
No	12	93.5	8	8.8	4	4.3	
**Sexual Orientation**							0.933
Straight/Heterosexual	124	67.8	61	67.0	63	68.5	
Gay/Lesbian	13	7.1	5	5.5	8	8.7	
Bisexual	34	18.6	18	19.8	16	17.4	
Other	12	6.6	7	7.7	5	5.4	
**Sex Assigned at Birth**							0.426
Male	41	22.4	22	24.2	19	20.7	
Female	142	77.6	69	75.8	73	79.3	
**Experienced Sexual Violence Before College**							0.822
Yes	84	45.9	45	48.9	43	47.3	
No	99	54.1	47	51.1	48	52.7	

**Notes:** p-value testing for differences between control and intervention group at baseline using Chi-Square

**Table 2:** Baseline Participant Reported Alcohol Use and Sexual Violence Measures

	All *n* = 183		Intervention *n* = 92		Control *n* = 91		
Outcome	Mean	SD	Mean	SD	Mean	SD	*p*
Past month # of alcohol use days (actual range 0–23)	6.30	4.41	6.24	4.12	6.32	4.74	0.677
Past month # of binge drinking days (actual range 0–13)	2.51	2.64	2.60	2.57	2.35	2.68	0.629
Knowledge of alcohol related SV (actual range 28–48)	37.47	3.57	37.43	3.49	37.51	3.67	0.761
Self-efficacy to obtain sexual consent (actual range 10–50)	42.10	7.45	41.85	8.08	42.35	6.81	0.763
Use of SV harm reduction strategies (actual range 18–53)	34.62	5.95	34.57	5.71	34.66	6.21	0.918
Past 3-month sexual violence experience (n/%)	88	48.1%	45	48.9%	43	47.3%	0.822

**Notes:** p-values provided for t-tests or Chi-square tests

**Table 3:** Regression Models for Alcohol Use and Sexual Violence-Related Variable, Post-Intervention and 3-Month Follow Up

	Post-Intervention				3-Month Follow Up			
Outcome	*b*	*(β)*	95% CI	*p*	*b*	*(β)*	95% CI	*p*
Past month # of alcohol use days (unadjusted)	−0.536	−0.057	(−1.929, 0.762)	0.467	−0.022	−0.003	(−1.150, 1.184)	0.981
Past month # of alcohol use days (adjusted)	−0.302	−0.032	(−1.652, 0.972)	0.662	−0.007	−0.001	(−1.157, 1.229)	0.989
Past month # of binge drinking days (unadjusted)	−0.5	−0.089	(−1.231, 0.270)	0.223	−0.319	−0.063	(−1.078, 0.549)	0.406
Past month # of binge drinking days(adjusted)	−0.371	−0.066	(−1.113, 0.391)	0.378	−0.311	−0.062	(−1.036, 0.461)	0.380
Knowledge of alcohol related SV (unadjusted)	0.53	0.174	(0.091, 0.908)	**0.022**	0.277	0.08	(−0.215, 0.747)	0.282
Knowledge of alcohol related SV (adjusted)	0.556	0.182	(0.132, 0.931)	**0.018**	0.253	0.073	(−0.241, 0.724)	0.321
Self-efficacy to obtain sexual consent (unadjusted)	0.709	0.131	(−0.010, 1.495)	0.077	0.625	0.111	(−0.198, 1.424)	0.143
Self-efficacy to obtain sexual consent (adjusted)	0.636	0.118	(−0.078, 1.418)	0.113	0.529	0.094	(−0.295, 1.345)	0.217
Use of SV harm reduction strategies (unadjusted)	0.284	0.065	(−0.330, 0.943)	0.383	0.371	0.083	(−0.263, 1.005)	0.277
Use of SV harm reduction strategies (adjusted)	0.331	0.076	(−0.288, 1.025)	0.321	0.461	0.103	(−0.225, 1.120)	0.770
	b	OR	95% CI	p	b	OR	95% CI	p
Past 3-month sexual violence experience (unadjusted)	−0.464	0.629	(0.349, 1.132)	0.122	−0.2	0.819	(0.455, .1.473)	0.505
Past 3-month sexual violence experience (adjusted)	−0.535	0.585	(0.299, 1.147)	0.118	−0.23	0.794	(0.416, 1.519)	0.486

**Notes:** Adjusted models control for sex assigned at birth, age, and history of sexual violence at baseline. Linear regression models include beta coefficients, with 95% bias-corrected and accelerated confidence intervals from 1,000 bootstrap samples. Logistic regression for sexual violence experience was conducted without bootstrapping. Statistically significant values noted in **bold**.

## Data Availability

The datasets during and/or analyzed during the current study available from the corresponding author on reasonable request.
